# Using twin-pairs to assess potential bias in polygenic prediction of externalising behaviours across development

**DOI:** 10.1038/s41380-025-02920-6

**Published:** 2025-02-19

**Authors:** Joanna K. Bright, Christopher Rayner, Ze Freeman, Helena M. S. Zavos, Yasmin I. Ahmadzadeh, Essi Viding, Tom A. McAdams

**Affiliations:** 1 Social, Genetic & Developmental Psychiatry Centre, Institute of Psychiatry, Psychology & Neuroscience, Kings College London, London, UK; 2 Department of Psychology, Institute of Psychiatry, Psychology & Neuroscience, Kings College London, London, UK; 3 Division of Psychology and Language Sciences, University College London, London, UK; 4 PROMENTA Research Center, Department of Psychology, University of Oslo, Oslo, Norway

**Keywords:** Genetics, Psychology, Predictive markers

## Abstract

Prediction from polygenic scores may be confounded by sources of passive gene-environment correlation (rGE; e.g. population stratification, assortative mating, and environmentally mediated effects of parental genotype on child phenotype). Using genomic data from 10 000 twin pairs, we asked whether polygenic scores from the most recent externalising genome-wide association study predict conduct problems, ADHD symptomology and callous-unemotional traits, and whether these predictions are biased by rGE. We ran regression models including within-family and between-family polygenic scores, to separate the direct genetic influence on a trait from environmental influences that correlate with genes (indirect genetic effects). Findings suggested that this externalising polygenic score is a good index of direct genetic influence on conduct and ADHD-related symptoms across development, with minimal bias from rGE, although the polygenic score predicted less variance in CU traits. Post-hoc analyses showed some indirect genetic effects acting on a common factor indexing stability of conduct problems across time and contexts.

## Introduction

Common externalising phenotypes, including conduct problems and attention-deficit/hyperactivity disorder (ADHD), are associated with adverse outcomes such as low academic achievement, social isolation and substance use disorders [1, 2]. More recently, callous-unemotional traits (CU) have been assessed concurrently and appear to index children with particularly severe behavioural problems [3, 4]. Previous research has shown that conduct problems, ADHD and CU traits are moderately to highly heritable [5, 6], share common aetiological influences [7] and can be predicted by polygenic scores extracted from genome-wide association studies (GWAS). Polygenic scores are increasingly used as tools to predict risk profiles [8] and it is hoped that in the future, polygenic scores will have clinical utility [9]. They can also be used by researchers as instrumental variables in causal inference analyses [10, 11].

It is increasingly understood that polygenic scores for complex traits may not simply index a person’s genetic liability. Instead, as genetic and environmental risks correlate, polygenic scores may also capture sources of passive gene-environment correlation (rGE), including population stratification, assortative mating, and the indirect genetic effects of parental genotype on child phenotype via parental behaviour (genetic nurture) [12–17]. Without accounting for such rGE, we cannot be sure of the true relationship between individuals’ direct genetic liability and subsequent behaviour.

Research has repeatedly shown that associations between polygenic scores and cognitive traits/ educational attainment are biased by rGE [13, 17, 18]. Symptoms related to conduct problems, ADHD, and CU traits have many features which make it plausible that polygenic prediction may also capture more than the direct genetic effects of a person’s genome on their phenotype. Previous research has suggested considerable assortative mating for externalising behaviours [19, 20] and the family environment is hypothesized to be a key contributor to externalising symptoms in children, so genetic nurture is a possibility [14]. Furthermore, externalising behaviours are strongly associated with socioeconomic status (SES) and educational attainment [21, 22], which are subject to population stratification, assortative mating, and parental effects [23]. Family-based models of externalising features have suggested some bias from rGE acting on externalising phenotypes. For example, associations between externalising polygenic scores and child ADHD are attenuated when parents’ genetic liability transmitted via the rearing environment was accounted for [24]. Similarly, single nucleotide polymorphism (SNP) heritability estimates for conduct disorder and ADHD symptoms were attenuated after accounting for the effects of parental genotype [14].

The potential benefit of using polygenic scores to elucidate variation in human behaviour is undermined if we do not systematically examine possible biases introduced by rGE. Using sibling pairs in genomic analyses allows estimation of direct genetic effects free from such biases, as siblings are matched for family environment (i.e. they share the effects of population stratification, assortative mating, and genetic nurture) [25]. Dizygotic (DZ) twin pairs are additionally matched for prenatal environment and time-variant factors. By using DZ twin pairs in our genomic analyses, we can thus account for some of the potential biases in polygenic prediction of our phenotypes of interest and get closer to capturing true effects of direct genetic influences within individuals. In short, by comparing polygenic prediction of phenotypes within-families (i.e., comparing prediction between DZ twins within a family) and between families (comparing prediction between family units in a sample), we can separate the direct genetic influence on a trait from environmental influences that correlate with genes.

As GWASs become larger, the variance explained by resultant polygenic scores tend to increase [26]. Consequently, there is more statistical power to detect portions of the variance attributable to passive rGE, not just direct genetic effects. The most recent externalising GWAS of one million individuals of European ancestry [27] provides an opportunity to use a polygenic score which captures genetic liability for externalising behaviours. Since its release, researchers have used the externalising polygenic score to investigate indirect genetic effects on externalising phenotypes using sibling/twin samples [27, 28] and parent-child units [28–30] To date such studies report minimal evidence for indirect genetic effects or genetic nurture in their samples. Here, we test for the presence of indirect genetic effects in a large longitudinal UK-based twin sample, to home in on genetic effects for externalising and related phenotypes as children grow up. Our study builds upon prior research by comparing multiple externalising-type phenotypes, testing across reporters and timepoints. We extend the developmental analyses from Tanksley et al. [28] using a larger sample, allowing us to consider each timepoint separately, towards a finer-grained understanding of developmental effects.

We investigated polygenic prediction for conduct problems, ADHD symptomology and CU traits, and the degree to which these predictions may be differentially biased by rGE. Where we found evidence for bias from indirect genetic effects in polygenic prediction, we tested whether measures of SES, neighbourhood deprivation, and parenting behaviours explained that bias, and whether these variables impacted estimates of direct genetic effects. We complemented our polygenic analyses of DZ twins with univariate twin analyses including both monozygotic (MZ) and DZ twins [31]. It has been suggested that genetic nurture effects may be captured in twin study estimates of the shared environment, as they are shared between twins and should promote similarity among both MZ and DZ twins [16, 32, 33]. We therefore considered that the magnitude of shared environmental estimates derived from twin analyses would be informative regarding the presence or absence of indirect genetic effects.

Our study addressed four key questions that have not been examined before. First, to what extent does the new polygenic score derived from a large-scale study of externalising-related behaviours index genetic liability for conduct problems, ADHD symptoms and CU traits respectively? Second, do the associations between the externalising polygenic score and these traits partly reflect environmental biases arising from sources of rGE? Third, does the degree of prediction and/or biases vary across development? Fourth, do environmental effects found in univariate twin models predict the presence of indirect genetic effects in genomic analyses? We hypothesised that the degree of prediction from the polygenic score to our phenotypes would be modest, in line with the currently observed magnitudes of variance accounted for by polygenic scores. We also hypothesised that prediction would be stronger for conduct problems and ADHD symptoms than for CU traits, given that prior research has indicated that CU traits have some level of genetic independence from broader externalising phenotypes [34–37]. Thirdly, we hypothesised that conduct problems would be most impacted by indirect genetic effects as prior research has typically reported higher estimates of shared environment for conduct problems than for ADHD symptoms or CU traits. We did not make specific hypotheses regarding the developmental effects, although we expected to find some variation, given previous research has shown differential polygenic prediction across developmental time periods [28].

## Methods

This study was preregistered on the Open Science Framework (https://osf.io/zh23d). Any additional non-preregistered analyses are considered exploratory and indicated in the text, with details in the Supplementary Materials.

### Sample

We used data from the Twins Early Development Study (TEDS; N~ = 10 000 families) [38], a cohort study of twins born in England and Wales between 1994 and 1997. We used available data from the whole sample when running univariate twin models, whereas for the genomic analyses we used a sub-sample of DZ pairs only (N = 7 063 pairs, 99.9% white [39]). Data were used from waves collected when twins were 4, 7, 9, 12, 16 and 21-years-old, rated by their parents, their teachers and self-reported at later ages. Supplementary Table 1 shows sample size and reporters at each wave. NB the TEDS sample at age 9 was half the size of other timepoints due to funding and operational constraints.

Twins were excluded from analyses following standard TEDS protocols, which removes those with serious medical conditions which hinders ability to participate, those who experienced extreme adverse perinatal circumstances, and those with missing essential background variables, including sex/gender or zygosity (https://teds.ac.uk/datadictionary/exclusions.htm). TEDS was approved by King’s College London’s ethics committee for the Institute of Psychiatry, Psychology and Neuroscience HR/DP-20/21-22060, and participant consent was collected at every stage of the TEDS data collection.

### Phenotypes

#### Conduct problems and ADHD symptoms

The Conduct Problems and Inattention-Hyperactivity subscales were taken from the Strengths and Difficulties Questionnaire (SDQ), a questionnaire aimed at identifying problem behaviours in children [40]. These were collected at twin ages 4, 7, 9, 12, 16 and 21 years of age.

#### Callous-unemotional traits

We created an index of callous-unemotional (CU) behaviours, from items collected at 7, 9, 12, and 16 years [41]. See Supplementary Methods for more information.

### Environmental covariates

#### Socioeconomic status (SES)

We used the TEDS index of SES, measured at first contact. The index is a composite score created from the standardised average of measures of mother and father employment levels, mother and father educational levels, and mother’s age on birth of first child [42].

#### Neighbourhood deprivation

The Index of Multiple Deprivation (IMD), is an index of neighbourhood deprivation created using participants’ post codes, giving a broader measure of wider environmental factors such as local levels of employment and education, crime rates, barriers to housing and living environment quality. More information on this score can be found on the UK government’s website (https://www.gov.uk/government/statistics/english-indices-of-deprivation-2010).

#### Parenting

We created a latent parenting factor from item-level measures of parenting available at each timepoint in the TEDS dataset. These consisted of ‘Parental Feelings’ and ‘Parental Discipline’ rated by the registered primary parent at ages 4, 7, 9 and 12. The factor was created using the lavaan package in R [43].

### Polygenic scores

Polygenic scores for externalising were created for DZ twin pairs using summary statistics from the current most recent GWAS of externalising liability in individuals of European ancestry (N = 10,45,957) [27]. Polygenic scores were computed using LDPred2 [44]. See Supplementary Methods for more information.

### Analyses

For each phenotype and time point, we ran two complementary analyses: within/between family polygenic score analyses and univariate twin models. All data quality control and statistical analyses were conducted in R version 4.3.1 [45].

#### Polygenic score analyses

To estimate the contribution of direct and indirect genetic effects on externalising traits we ran two linear regression models in the DZ twin sample for each outcome at each timepoint (conduct problems, ADHD, and CU traits) [13].

1 $${{EXT}}_{{ij}}={\alpha }_{0}0+{{{\rm{\beta }}}}\left({{{{\rm{PGS}}}}}_{{{{\rm{ij}}}}}\right)+{{{{\rm{Z}}}}}_{{{{\rm{ij}}}}}$$

2 $${{EXT}}_{{ij}}=\,{\alpha }_{0}0+{{{\rm{\beta }}}}{{{{\rm{Within}}}}}_{{{{\rm{EXT}}}}}\left({{{{\rm{PGS}}}}}_{{{{\rm{ij}}}}}-\bar{{{\mbox{PGS}}}_{{{{\rm{j}}}}}}\right)+{{{\rm{\beta }}}}{{{{\rm{Between}}}}}_{{{{\rm{EXT}}}}}\left(\bar{{{\mbox{PGS}}}_{j}}\right)+{{{{\rm{Z}}}}}_{{{{\rm{ij}}}}}$$

The first model estimated the population-level effect and included the polygenic score as a fixed effect (Eq. 1, where for twin *i* in twin-pair *j*, EXT denotes the externalising outcome, and PGS is externalising polygenic score). The second model included the within-family and between-family polygenic scores as separate fixed effects (Eq. 2) [13]. Here, the between family score is simply the family-based (twin-pair-based) mean polygenic score. The within-family polygenic score is the between family polygenic score subtracted from the individual’s polygenic score (twin *i*’s polygenic score minus their twin-pair’s mean score, ${{{{\rm{PGS}}}}}_{{{{\rm{ij}}}}}-\bar{{{\mbox{PGS}}}_{{{{\rm{j}}}}}}$) and between-family terms (the twin-pair’s polygenic score, $\bar{{{\mbox{PGS}}}_{{{{\rm{j}}}}}}$). We included age, sex, age*sex, genotyping platform and the first 10 ancestry principal components in the Z term, as covariates in the models. Individuals with missing data were omitted from each regression analysis.

Extracted effects of the polygenic scores from these regressions were used to calculate the indirect genetic effect by subtracting the direct genetic estimate (within-family effect) from the population estimate (between-family effect).

We computed bootstrapped standard errors and bias corrected confidence intervals for all effect estimates (population, direct and indirect), using the boot() function in R [46], with 10,000 replications.

#### Examining potential sources of indirect genetic effects

Where indirect genetic effects were found, we ran analyses controlling for SES, neighbourhood deprivation or parenting behaviours accounted for these indirect genetic effects.

##### Univariate twin models

We ran univariate twin models at the same timepoints as the polygenic score analyses to evaluate whether derived aetiological estimates give any insight into whether indirect genetic effects may be present in associations between polygenic scores and our phenotypes. Specifically, it has been noted that indirect genetic effects share much in common with the shared environment, so we asked whether non-zero estimates of shared-environment influence predicted non-zero estimates of indirect genetic effect. Univariate twin models were applied to decompose phenotypic variation into additive genetic (A), dominant genetic (D) or shared environmental (C), and non-shared environmental (E) variance components, known as ADE or ACE models respectively (see Supplementary Fig. 1) [31]. Analyses were run using the OpenMx R package [47], using Full Information Maximum Likelihood (FIML) estimation. All outcomes were adjusted for covariates (age, sex and sex*age). Contrast effects, where parents of non-identical twins contrast their twins and overestimate their differences, have been shown in parent-reported ADHD for dizygotic twins [48, 49]. To control for this phenomenon, we included a sibling-interaction term into the univariate twin models for parent-reported ADHD symptoms.

#### Common factor analyses (not pre-registered)

After examining our data, we ran exploratory, post-hoc analyses using common factor scores for each phenotype computed from all available items across time and reporters. These factors thus capture stability across time and reporter. Although we did not pre-register these analyses, we believe these are important to include to contextualise our main findings, since genetic signal tends to be greater on indices of behavioural stability across time/contexts than on cross-sectional time/context specific measures, as common factor scores reduce measurement error, compared measures specific to a single timepoint or context [12, 48]. We reasoned that running such analyses might further increase our power for detecting direct and indirect genetic effects. Common factor scores (Supplementary Figs. 2, 3) were computed from item-level data using the cfa() function in the lavaan package for R [43], using FIML estimation. We also computed reporter- and age-specific factor scores to test whether effects varied by reporter or developmental stage. These additional results explore the extent to which focussing only on time/context specific analyses reduces our ability to detect genetic signal, and thus to distinguish direct from indirect genetic effects.

## Results

### Cross-sectional polygenic score analyses

**Fig. 1:**
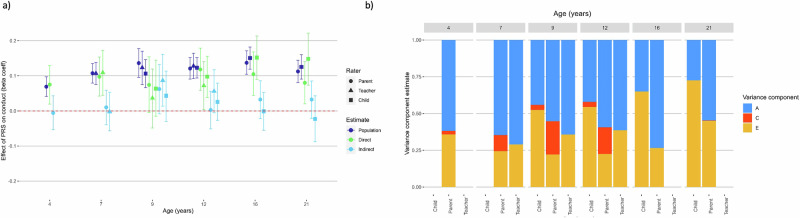
Polygenic score analysis and twin models for conduct problems. **a** Estimating population-level, direct and indirect genetic effects of externalising PRS. Beta coefficient estimates of population-level prediction of externalising PRS for conduct problems, alongside estimates of direct and indirect genetic effects, reported across developmental timepoints and reporter. **b** Estimating univariate ACE twin models for conduct problems. Estimates of the contribution of additive genetic effects (A), common environmental influences (C) and unique environmental influences (E) in the variance of conduct problems. Each univariate twin model was repeated at each timepoint, and for parent, teacher, and self-reports.

**Fig. 2:**
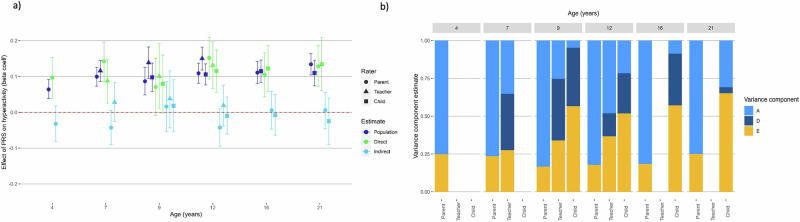
Polygenic score analysis and twin models for ADHD symptoms. **a** Estimating population-level, direct and indirect genetic effects of externalising PRS. Beta coefficient estimates of population-level prediction of externalising PRS for ADHD symptoms, alongside estimates of direct and indirect genetic effects, reported across developmental timepoints and reporter. **b** Estimating univariate ADE twin models for ADHD symptoms across development and reporter. Estimates of the contribution of additive genetic effects (A), dominant genetic effects (D) and unique environmental influences (E) in the variance of hyperactivity problems. Each univariate twin model was repeated at each timepoint, and for parent, teacher, and self-reports. D was dropped from the parent models as it was not significant once sibling interaction terms were included.

The externalising polygenic score predicted 0.1–2.3% of the variance in conduct problems, ADHD symptoms and CU traits. We found a similar pattern of results for conduct disorder and ADHD symptomology (see Figs. 1a and 2a). The polygenic score predicted an average of 1.4% of the variance in the phenotype for conduct problems and 1.3% for ADHD symptoms. There was an increase in prediction for parent-reported conduct problems over development, confidence intervals were non-overlapping for age 4 and age 16 (prediction increased from 0.6% variance explained at age 4, to a peak of 1.9% at age 16). Similarly, prediction rose from 0.5% to 1.8% of the variance in parent-rated ADHD symptoms from age 4 to 21 (confidence intervals non-overlapping). At each timepoint, there was no significant difference in polygenic prediction between reporters for conduct problems or ADHD symptoms. Polygenic prediction of conduct problems and ADHD symptoms was almost wholly due to direct genetic effects at all ages, i.e. there was no evidence for a role of indirect genetic effects on these externalising outcomes. The only exception to this was for teacher-reported conduct problems at age 9, we did find significant indirect genetic effects (Fig. 1a).

**Fig. 3:**
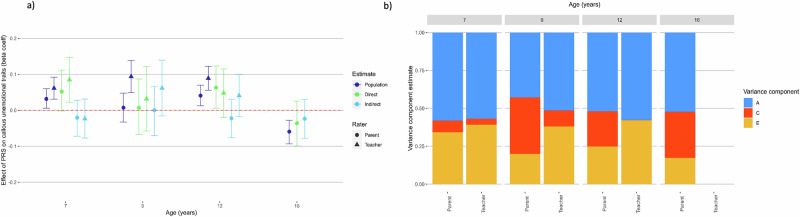
Polygenic score analysis and twin models for callous-unemotional traits. **a** Estimating population-level, direct and indirect genetic effects of externalising PRS. Beta coefficient estimates of population-level prediction of externalising PRS for an index of callous-unemotional traits, alongside estimates of direct and indirect genetic effects, reported across developmental timepoints and reporter. **b** Estimating univariate ACE twin models for callous-unemotional traits across development and reporter. Estimates of the contribution of additive genetic effects (A), common environmental influences (C) and unique environmental influences (E) in the variance in an index of callous-unemotional traits. Each univariate twin model was repeated at each timepoint, and for parent and teacher reports.

The polygenic score predicted less of the variance in CU traits, averaging 0.4% of variance explained (Fig. 3a). The confidence intervals for polygenic prediction of parent and teacher reported CU traits were overlapping at age 9. There was no change in magnitude of prediction between ages 7 and 12, although at age 16 the polygenic score had a negative association with parent-reported CU traits. We did not find significant direct or indirect genetic effects on CU traits once we broke the polygenic prediction down into within-family and between-family effects.

### Cross-sectional twin analyses

The results from twin models are shown in Figs. 1b–3b. Shared environmental effects (C) were estimated as significant for parent reported conduct problems at age 7 (11% of variance), age 9 (23%) and age 12 (18%). MZ twin correlations were more than double DZ twin correlations, so an ADE model (D = dominant genetic effect) was appropriate, and there was no significant contribution of D to the variance in parent-reported ADHD symptoms and so we dropped D from these models. See Supplementary Results for a more detailed report of results from twin models. For CU traits, estimates of C were significant for parent reported CU at all ages: explaining between 7–37% of variance. C also significantly explained 11% of the variance in teacher-reported CU traits at age 9. However, in polygenic analyses significant indirect genetic effects were not detected for any of these variables, suggesting that significant shared environment estimates were not predictive of indirect genetic effects.

### Examining potential sources of indirect genetic effects

**Fig. 4:**
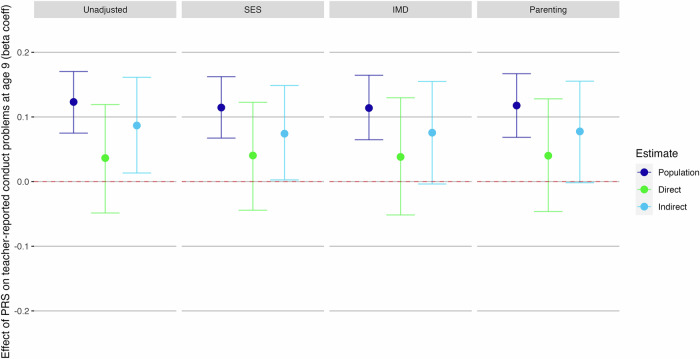
Investigating impact of socioeconomic status, neighbourhood deprivation or parenting variables on direct and indirect genetic effects of externalising PRS on teacher-reported conduct problems at age 9. Beta coefficient estimates of population-level prediction of externalising PRS, alongside estimates of direct and indirect genetic effects, for teacher-reported conduct problems at age 9, where we found significant indirect genetic effects. Socioeconomic status (SES) was measured at first contact and comprises measures of parent employment, education, and age of mother on first birth. The Indices of Multiple Deprivation (IMD) decile score uses census data matched with participants post codes, giving a broader measure of wider environmental factors such as local levels of employment and education, crime rates, barriers to housing and living environment quality. The parenting analyses used a latent factor created from ‘Parental Feelings’ and ‘Parental Discipline’ rated by parent at ages 4, 7, 9 and 12.

As we found indirect genetic effects acting on polygenic prediction of teacher-reported conduct problems at age 9, we re-ran these models controlling for SES, neighbourhood deprivation or parenting-related factors to assess whether any potential indirect genetic effects were captured by these covariates (Fig. 4). Estimates of direct genetic effects were minimally impacted by including these covariates. Further, we found that controlling for either neighbourhood deprivation or parenting behaviours entirely accounted for the indirect effect (Fig. 4; 95% confidence intervals included zero). When controlling for SES, the 95% confidence interval for the indirect genetic effect did not include zero, although the lower interval was very close, at 0.003 (Fig. 4).

### Post-hoc common factor analyses: stability across time and contexts

**Fig. 5:**
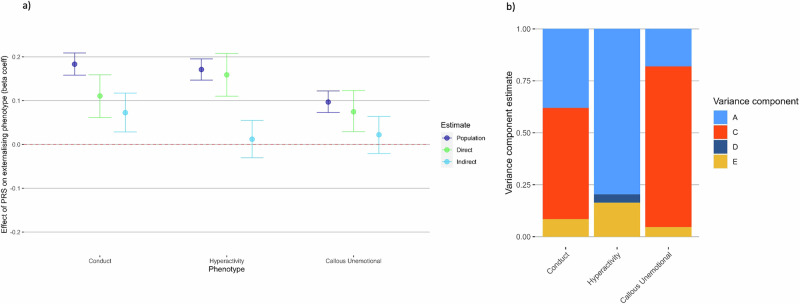
Polygenic score analysis and twin models for common factor scores. Factor scores were created using common factor analysis in lavaan, extracting stability for each phenotype from measures across all timepoints and reporter. **a** Estimating population-level, direct and indirect genetic effects of externalising PRS. Beta coefficient estimates of population-level prediction of externalising PRS alongside estimates of direct and indirect genetic effects, for common factor scores created for conduct problems, ADHD symptoms and callous-unemotional traits. **b** Estimating univariate ACE/ADE twin models for common factor scores for each phenotype. Estimates of the contribution of additive genetic effects (A), common environmental influences (C) or dominant genetic effects (D), and unique environmental influences (E) in the variance of each factor.

We extracted common factor scores from item-level data for each phenotype to index trait stability across time and reporter (Fig. 5a). The polygenic score predicted 3.4% of variance in the conduct problems factor, 2.9% of variance in the ADHD symptoms factor and 1.0% of variance in the CU traits factor. Using these factors in the within- and between-family regression models, we found significant indirect genetic effects on conduct problems, which made up 40% of the total prediction. We ran a sensitivity analysis removing age 9 items from the factor score, as this timepoint contained half the sample size of the rest of the ages (Supplementary Fig. 7). The finding of a significant indirect genetic effect remained in this sensitivity analysis. For ADHD symptoms and callous-unemotional traits, the prediction was wholly due to direct genetic effects. Further analyses showed that these results were consistent when stratified by reporter or timepoint (See Supplementary Information, Supplementary Figs. 4a and 5a). When using a common factor score for callous-unemotional traits, we were able to detect some population and direct genetic effects, whilst indirect genetic effects remained non-significant.

**Fig. 6:**
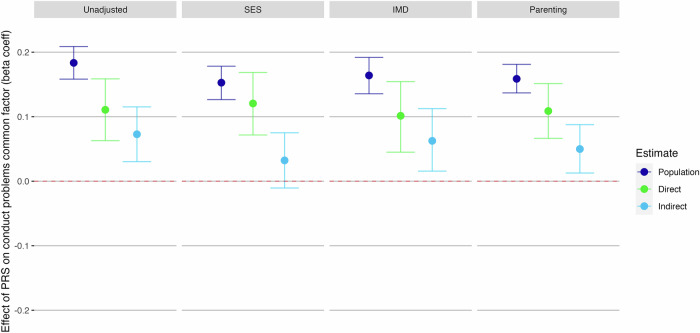
Investigating impact of socioeconomic status indices or parenting variables on direct and indirect genetic effects of externalising PRS on the common factor score for conduct problems. Socioeconomic status (SES) was measured at first contact and comprises measures of parent employment, education, and age of mother on first birth. The Indices of Multiple Deprivation (IMD) decile score uses census data matched with participants post codes, giving a broader measure of wider environmental factors such as local levels of employment and education, crime rates, barriers to housing and living environment quality. The parenting analyses used a latent factor created from ‘Parental Feelings’ and ‘Parental Discipline’ rated by parent at ages 4, 7, 9 and 12.

Where we found indirect genetic effects acting on polygenic prediction of stability (in conduct problems), we re-ran these models controlling for SES, neighbourhood deprivation or parenting-related factors (Fig. 6). Estimates of direct genetic effects were minimally impacted by including these covariates (attenuation of between 2–11% of the effect sizes found in uncontrolled analyses). For the common factor score of conduct problems, we found that controlling for SES reduced the indirect genetic effect to zero (Fig. 6). Neither the index of neighbourhood deprivation nor parenting behaviours impacted the significance of the indirect genetic effects on the common factor score for conduct problems, however they did account for the indirect genetic effect on two out of four within-time factors. Controlling for parenting led to a non-significant effect of the polygenic score on within-twin and within-teacher factors.

When using a common factor score in twin models (Fig. 5b), we found a much larger presence of C for conduct problems (80%) and CU traits (77%) than in the cross-sectional analyses. When we broke down each factor into within-reporter factors (Supplementary Figs. 4b and 5b), we can see that this large estimate of C was driven by parent-reports, for both conduct problems and CU traits. For ADHD symptoms, A accounted for the majority of variance, with minimal influence of D or E. Although significant C estimates in twin analyses did not reliably predict the presence of indirect genetic effects, where indirect genetic effects were detected in polygenic score analyses (i.e. for conduct problems), C was consistently significant in twin analyses.

## Discussion

Polygenic scores hold great potential for better understanding the developmental aetiology of psychiatric and behavioural problems, including externalising phenotypes. However, studies that examine associations between polygenic scores and such outcomes should also investigate possible sources of passive gene-environment correlation (rGE). Passive rGE may confound associations between individuals’ direct genetic liability and subsequent behaviour, so before we conclude that polygenic scores represent direct genetic risk, this assumption should be examined. We tested for indirect genetic effects on associations between a polygenic score for externalising (derived from a GWAS of over one million people of European Ancestry [27]; and parent-, teacher- and self-reported measures of conduct problems, ADHD symptoms, and callous-unemotional (CU) traits by using dizygotic twin pairs from a developmental twin cohort. Findings from our main, pre-registered analyses suggested that this externalising polygenic score is a good index of direct genetic influence on conduct and ADHD-related symptoms across development, with minimal bias from rGE. For CU traits, the polygenic score predicted less variance.

In cross-sectional analyses we found no statistically significant indirect genetic effects on either conduct problems or ADHD-related symptoms at any age or for any reporter, except teacher-reported conduct problems at age 9. This aligns with previous research, which describes minimal attenuation of associations between externalising polygenic scores and externalising phenotypes when controlling for potential passive rGE confounding [27–29]. It is possible that the indirect genetic effects found on teacher-reported conduct problems at age 9 is a spurious finding: the TEDS sample at age 9 was half the size of other timepoints, with non-significant direct genetic effects. For parent and child reported conduct problems at age 9, neither direct nor indirect effects appeared significant, suggesting we lacked the power to accurately decompose the signal in the data available at this time point. Overall, in this sample of developing children, the externalising polygenic score served as a good marker of direct genetic influence on conduct problems and ADHD symptoms, seemingly unbiased by indirect genetic effects such as population stratification and genetic nurture.

The externalising polygenic score provided a much lower prediction of CU symptoms than conduct problems or ADHD symptoms. Given the externalising polygenic score used in this study was derived using phenotypes that were more closely related to conduct problems and ADHD than CU traits, it is perhaps not surprising that the prediction was less strong. Twin data also indicates only moderate overlap between heritability of conduct problems/ADHD symptoms and CU traits [35–37]. Therefore, our findings are line with the notion that although CU traits are associated and share genetic risk factors with conduct problems and ADHD symptoms, they are also influenced by genes not shared with these externalising phenotypes [7].

When looking across development, from the ages 4 to 21 years, we saw a small increase in population-level polygenic prediction of conduct problems and ADHD symptoms. This may depict the genes involved in the presentation of these externalising symptoms having a stronger influence later in childhood and early adulthood. However, it is important to consider that the polygenic scores used here were created from a GWAS made from meta-analysed GWAS samples capturing a range of ages from children to much older adults. Previous research has shown that genetic influences on the intercept and slope of conduct problems, ADHD symptomatology, and CU traits are substantial, but largely non-overlapping [41, 50, 50]. This means the genes impacting the initial risk for developing these phenotypes and those impacting their developmental course appear to be at least partially distinct. Therefore, as our genetic index was not created specifically for young children, the prediction may not be equally as good in young children as compared to early adulthood, due to developmental genetic effects.

We had anticipated that associations between an externalising polygenic score and conduct problems, ADHD symptoms and CU traits could be subject to bias from passive rGE. However, our main analyses did not find evidence for this. One possible explanation for this may be that the genes picked up in the GWAS are not the same genes that drive passive rGE. The polygenic score accounted for a maximum of 2.3% of conduct problems, ADHD symptom and CU trait variance, which suggests there are genetic effects not captured in this polygenic score, which are yet to be found.

Whilst there was limited evidence for indirect genetic effects via passive rGE, evocative/active rGE may still be operating and acting as a route for direct genetic effects to be expressed [51]. For example, a child with a genetic propensity for conduct problems may evoke more authoritarian parenting behaviours in a caregiver, or actively seek a peer group who engage in rule-breaking behaviours, and these genetic influences on the environment may in turn elicit stronger conduct problems from the child. These pathways describe direct genetic effects (the effects of an individual’s DNA on their phenotype unconfounded by passive rGE) even though these direct effects are mediated by the environment.

We ran exploratory post-hoc analyses where we examined whether a different picture would emerge if we indexed stability on the externalising traits using common factor analyses across time and reporter. These yielded increased prediction from polygenic scores, and we also found a significant indirect genetic effect which accounted for 40% of the population-level prediction for conduct problems. This indirect genetic effect on conduct problems remained using within-reporter across-time and within-timepoint across-reporter common factors. These time and/or context stable indices capture something more trait-like than measures at a single time-point from a single reporter, reducing error and increasing statistical power. In our analyses, it seems that indirect genetic effects are present (or at least large enough to detect in the present sample) for conduct problems, but only when focussing on stable variance. Similarly, with the increased power of using a common factor score for CU traits, we were able to detect some direct genetic effects, whilst indirect genetic effects remained non-significant.

Research has suggested that stable sources of environmental influence on child behaviour such as SES and parenting behaviours may correlate with genetic risk and so contribute to indirect genetic effects [17]. Therefore, we ran analyses controlling for influences of SES, neighbourhood deprivation and parenting behaviours where we found significant indirect genetic effect; i.e. teacher-reported conduct problems at age 9 and common factor score analyses for conduct problems. For teacher-reported conduct problems at age 9, we found that controlling for neighbourhood deprivation or parenting behaviours accounted for the indirect effect. The indirect genetic effect on the common factor for conduct problems was no longer present once SES was included in the model, whilst the direct genetic effect showed minimal attenuation. This is encouraging for researchers who want to work with these polygenic scores to predict conduct problems but who do not have twin or family data, as our results suggest that including a measure of individual-level SES can account for bias from indirect genetic effects without impacting the estimation of direct genetic influence. It is notable that unlike conduct problems, we did not find indirect genetic effects for ADHD symptoms or CU traits, even when focussing on latent indices of stability. This was despite the conduct problems and ADHD symptoms having similar SNP heritability, suggesting similar power to detect indirect genetic effects in the ADHD analyses. This may point to differing influence of parent- and family-related factors on the stability of conduct problems and ADHD symptoms, aligning with prior published twin research which often describes shared environmental influences on conduct problems, but less often finds such influences for ADHD or CU traits [35, 50, 52]. The polygenic score predicted less variance in CU traits than conduct problems, so further investigation is needed with a more appropriate polygenic score for CU traits to determine whether we are capturing a true direct genetic effect or if there is an issue of power to detect indirect effects.

Finally, we hypothesised that indirect genetic effects in the polygenic analysis may relate to estimates of shared environmental influence (C) in the twin models. We found very little evidence for indirect genetic effects, however for those indirect genetic effects that we did find, C was also present in the complementary twin analyses. However, the reverse was not true: shared environmental effects did not predict indirect genetic effects. It is worth considering that indirect genetic effects will not always be shared by twins and so may contribute to non-shared environmental influences (E). For example, differential experience of parenting behaviours that are associated with the parent’s genotype would load onto E in a twin model. That said, we note that there was very minimal evidence for E in our twin analyses using the conduct problems factor score, where we did find evidence for indirect genetic effects.

We note some limitations to our analyses. The sample at age 9 is half the size of other time-points, and this is where we found the only significant indirect genetic effect in the cross-sectional analysis. The mismatch in sample size limits comparison to other timepoints and interpretation of the significant finding. Secondly, the GWAS that was used to derive our polygenic scores includes phenotypes more strongly associated with conduct problems and ADHD symptoms. Therefore, the polygenic scores used may not be entirely suitable for predicting direct genetic liability for CU traits. Finally, for genomic analysis, the samples used in the GWAS were restricted to individuals with a European ancestry [27], as were the individuals included in genotyping in TEDS (99.9% white). Thus, we cannot generalise these results to non-European ancestry populations. Future research should test this polygenic score in other samples to confirm whether these results replicate across populations.

Within-family designs aim to reduce bias in direct genetic estimates; however, they are not without their own methodological concerns. Research has shown that family-fixed effects models can bias within-family estimates of direct genetic effects towards zero [53], whilst overestimating population-level (between family) effects. Whilst the non-zero estimates for direct genetic effects shown across most analyses suggest that such results are not completely due to methodological biases, we can only give limited interpretation to findings of no direct genetic effects, such as with CU traits in our analyses. We also recognise criticism of the sibling design; whereby indirect genetic effects may be biased by varying confounding effects (in both positive and negative directions). This bias can be difficult to measure within sibling designs, leading to an inaccurate estimate of direct genetic effects [54]. Triangulation across different family-based designs, such as sibling GWAS or intergenerational analyses will help to elucidate various routes of direct and indirect genetic effects. For example, the inclusion (or imputation) of parental genotypes can improve ability to estimate the contribution of direct and indirect genetic effects on a phenotype [55]. Research using parent-child trios has found no evidence for genetic nurture on externalising behaviours [28, 29], suggesting that findings of limited indirect genetic effects are consistent across study designs.

Our study offers converging evidence to a growing body of research that takes advantage of family-based samples to systematically evaluate the predictive power of the latest externalising polygenic score. As with other studies, we confirmed the suitability of the externalising polygenic score in explaining variation in conduct problems, ADHD symptoms and CU traits across development, with little evidence for bias from passive rGE. A particular strength of this study was the use of multiple study designs to draw inference regarding direct and indirect genetic effects. No prior study using polygenic scores has focused on these three phenotypes simultaneously and we took a novel developmental approach, using the same measures at multiple timepoints. We found robust evidence for direct genetic effects of the best-powered polygenic score for externalising on conduct problems and ADHD symptoms, that appeared consistent across reporters. We also demonstrated a novel finding of indirect genetic effects on the stability of conduct problems, which warrant further investigation. The externalising polygenic score predicted less variance in CU traits, suggesting a partially distinct, genetic aetiology for CU traits. Our study highlights the importance of considering the measures, constructs and analyses we use, as we seek to understand developmental risk for externalising problems and CU traits and to prevent these adverse outcomes.

## Supplementary information

Supplementary Information (Methods, Results, Tables, Figures)

## Data Availability

The datasets generated during and/or analysed during the current study are not publicly available as the consent given by the participants does not allow for data storage on an individual level in repositories or journals. Access to datasets requires approval from the relevant access committees at Twins Early Development study (TEDS). Researchers who would like to access the datasets used herein should contact the lead or corresponding authors.

## Code availability

Code used for these analyses can be accessed at https://osf.io/vcdnx/.
