# Supplementary material for: Using twin-pairs to assess potential bias in polygenic prediction of externalising behaviours across development
Source: Mol Psychiatry. 2025 Feb 19;30(7):3129–37. doi: 10.1038/s41380-025-02920-6 (PMC12185310; doi:10.1038/s41380-025-02920-6)
Supplement: Supplementary file 1 — Supplementary Information (Methods, Results, Tables, Figures) [file 41380_2025_2920_MOESM1_ESM.docx]

**Supplementary information**

**Supplementary Methods**

***Callous Unemotional index creation***

CU traits at ages 7, 9 and 12 years old were assessed by a seven-item scale, as shown in previous work[1]. CU scores were composed of four Strengths and Difficulties Questionnaire items which cover prosocial behaviour (reverse-scored to capture anti-sociality; i.e. ‘Considerate of other people’s feelings’, ‘Helpful if someone hurt’, ‘Have at least one good friend’ and ‘Kind to younger children’) and three Antisocial Process Screening Device (APSD)[2]: CU subscale items (i.e. ‘Does not show feelings or emotions’ ‘Guilty when does something wrong (reverse-scored)’ and ‘Concerned to do well (reverse-scored)’). Each item was rated on a 3-point Likert scale (0 = Not true,1= Somewhat true and 2 = Certainly true). The CU score at age 16 was also computed from the same four SDQ items, and three items from the Inventory of Callous–Unemotional Traits (ICU)[3] with the same item content than the APSD CU subscale, but rated on a 4-point Likert scale ranging from 0 (Not at all true) to 3 (Definitely true). When creating the composite score for age 16, to adjust score range of the ICU to the APSD, we applied a linear transformation[4] to the ICU’s scaling changing it from 0 to 3, to 0 to 2 to match the APSD. These three item scores were multiplied by two thirds before creating the composite score in CU traits for age 16. All scores were regressed on age and sex prior to analyses in this study.

***Polygenic score generation***

To calculate polygenic scores, we used a Bayesian approach to polygenic score calculation, implemented in the software LDpred2[5]. In this method, a posterior effect size is calculated for each single SNP that is present in both the GWA study summary statistics and the target genotype sample. To calculate the posterior effect size, the original summary statistic effect size estimates are adjusted based on two factors: (a) the relative influence of a SNP given its level of LD with surrounding SNPs in the target sample (here TEDS), and (b) a prior on the effect size of each SNP. To account for LD, we set the radius to a 2 megabase window. The effect size prior depends on the SNP-heritability of the discovery (i.e., GWA study) trait and an assumption on the fraction of causal markers believed to influence the discovery trait. Using the prior, the beta effect sizes are reweighted such that the effects are spread out among the SNPs across the whole genome in proportion to the LD present among these SNPs. In the creation of the polygenic scores, summary stats were filtered to remove (1) rare SNPs (minor allele frequency < 0.005), (2) SNPs with an IMPUTE imputation quality (INFO) score < 0.9, (3) SNPs that could not be mapped to or had discrepant alleles with the reference panel and (4) otherwise low-quality variants. Thus, 6,170,305 SNPs were included analysis. Finally, all trait-associated alleles were counted (0, 1, or 2 for each SNP), weighted by the posterior SNP effect size obtained through LDpred, and summed across the genome to calculate a polygenic score for everyone in TEDS.

**Supplementary Results**

***Twin models***

For conduct problems, additive genetic (A) effects explained of 25-75% of the variance in conduct problems, 5-80% in ADHD symptoms and 43-58% in CU traits. Common environmental influences (C; influences which make twins more similar for a trait), were largely non-significant for conduct problems across timepoints and reporters, except for parent reported conduct problems at ages 4-12, where C estimates ranged from 5-25%. For callous unemotional traits, C explained 10-30% of the variance. When modelling ADHD symptoms, MZ twin correlations were more than double DZ twin correlations, so an ADE model (D = dominant genetic effect) was appropriate (Figure 5). There was no significant contribution of D to the variance in parent-reported ADHD symptoms and so we dropped D from these models. Applying univariate twin models to common factor scores indexing shared variance across development and reporter, we found that C explained most of the variance in conduct problems (53%) and callous unemotional traits (77%), whereas A explained the majority of variance for ADHD symptoms (80%) (Figure 11). Using factors split by reporter showed that the large effect of C on conduct problems and callous unemotional traits was largely driven by parent-reported measures (Figure 12). Timepoint specific factors showed that A had the most influence on the variance across all three phenotypes (Figure 13)

**Post-hoc polygenic analyses**

Analyses of common factors indexing stability stratified by reporter or timepoint showed that these results were consistent across reporter (Supplementary Figure 4a) and timepoints (Supplementary Figure 5a), where significant indirect effects were found on across time within-reporter (child = 30%, parent = 39% and teacher = 26% of total prediction), and across reporters within-time-points (age 7 = 56%, age 9 = 35%, age 12 = 36%, age 16 = 32%, age 21 = 28% of total prediction) but not for ADHD symptoms or callous-unemotional traits (with the one exception of significant indirect genetic effect on the factor for callous-unemotional traits at age 12).

***Supplementary Table 1: Sample sizes for the whole sample (for twin models), and dizygotic (DZ) twin sample for polygenic risk score analyses, across timepoints and reporters.*** Complete cases are the number of individuals with data at all timepoints.

***
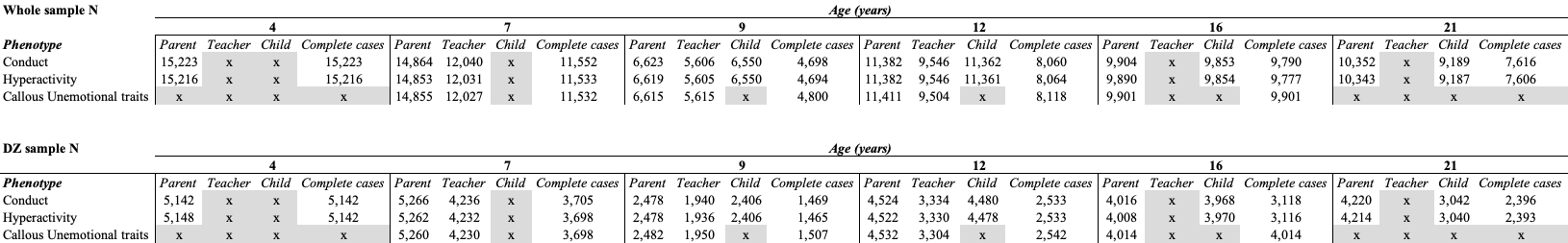
***

***Supplementary Figure 1: Univariate Twin model.***

Decomposes variance in a trait into additive genetic effects (A), common environmental influences (C) and unique environmental influences (E). Monozygotic (MZ) twins have a correlation of 1.0 for A, whereas dizygotic (DZ) twins have a correlation of 0.5. In this model, all twin pairs have a correlation of 1.0 for C.

***
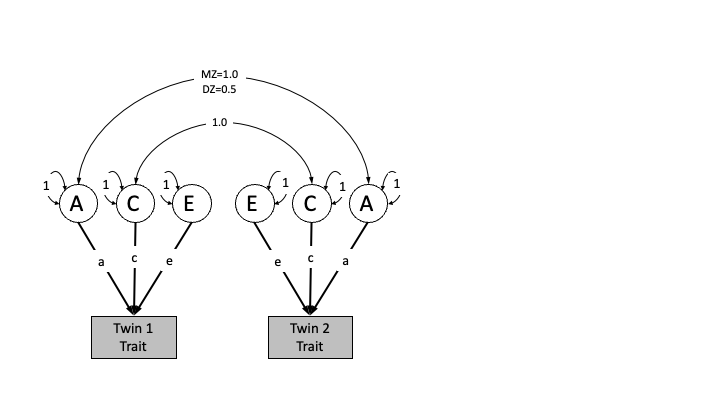
***

***Supplementary Figure 2: Latent factor for conduct and hyperactivity problems.***

Factor created from item-level measures of externalising across all time points and reporters. We also created latent factors stratified by reporter or timepoint separately.

**
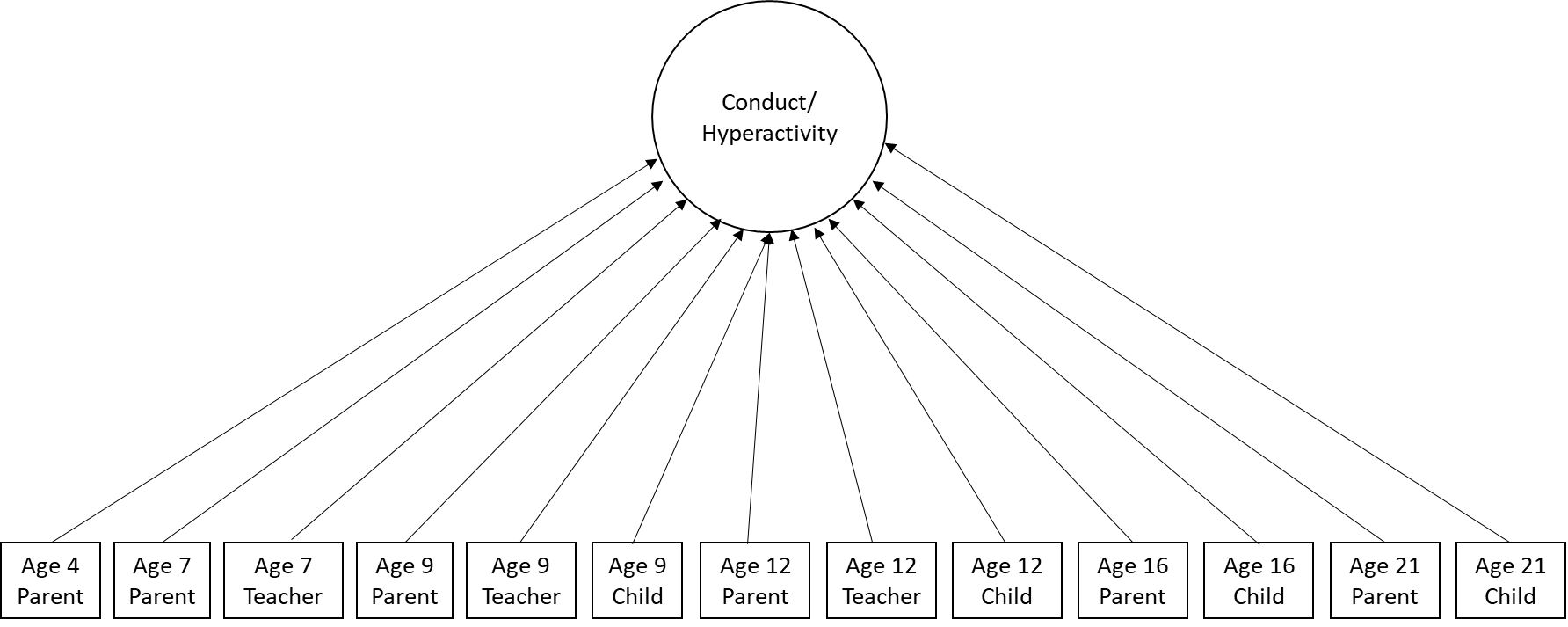
**

***Supplementary Figure 3: Latent factor for callous unemotional traits.***

Factor created from item-level measures of callous-unemotional traits across all time points and reporters. We also created latent factors stratified by reporter or timepoint separately.

**
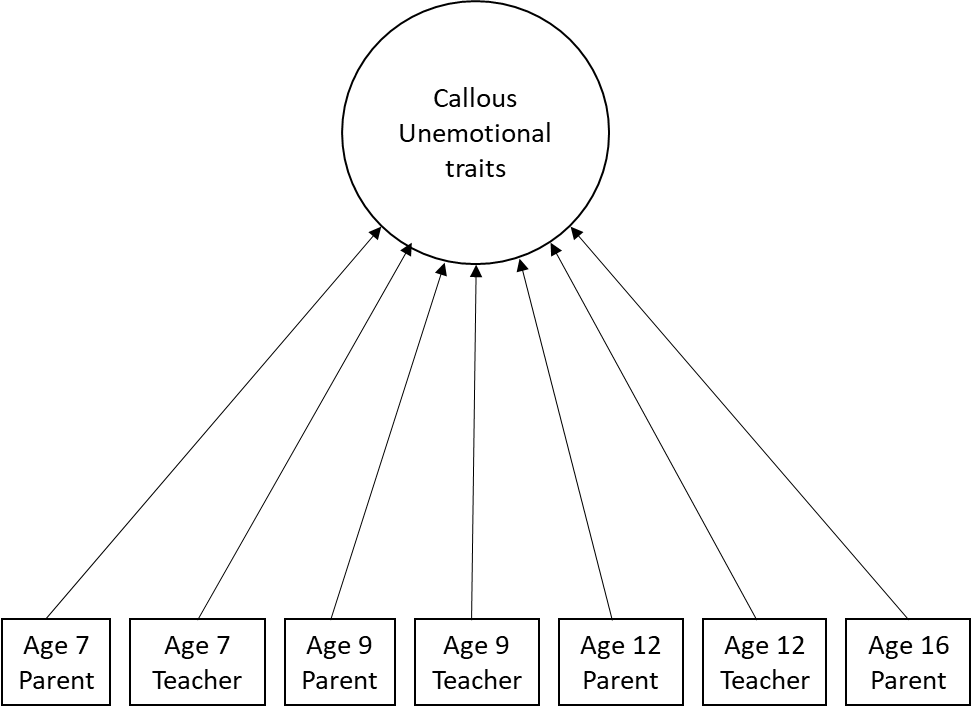
**


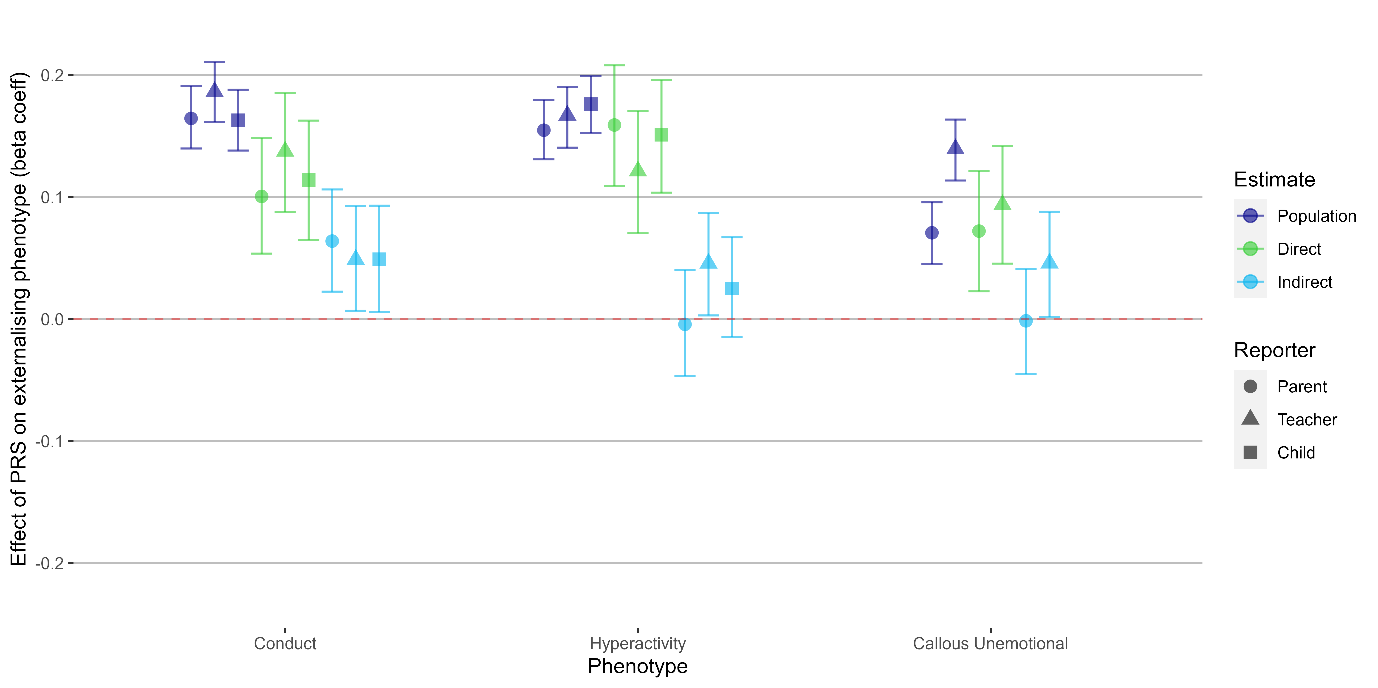
***
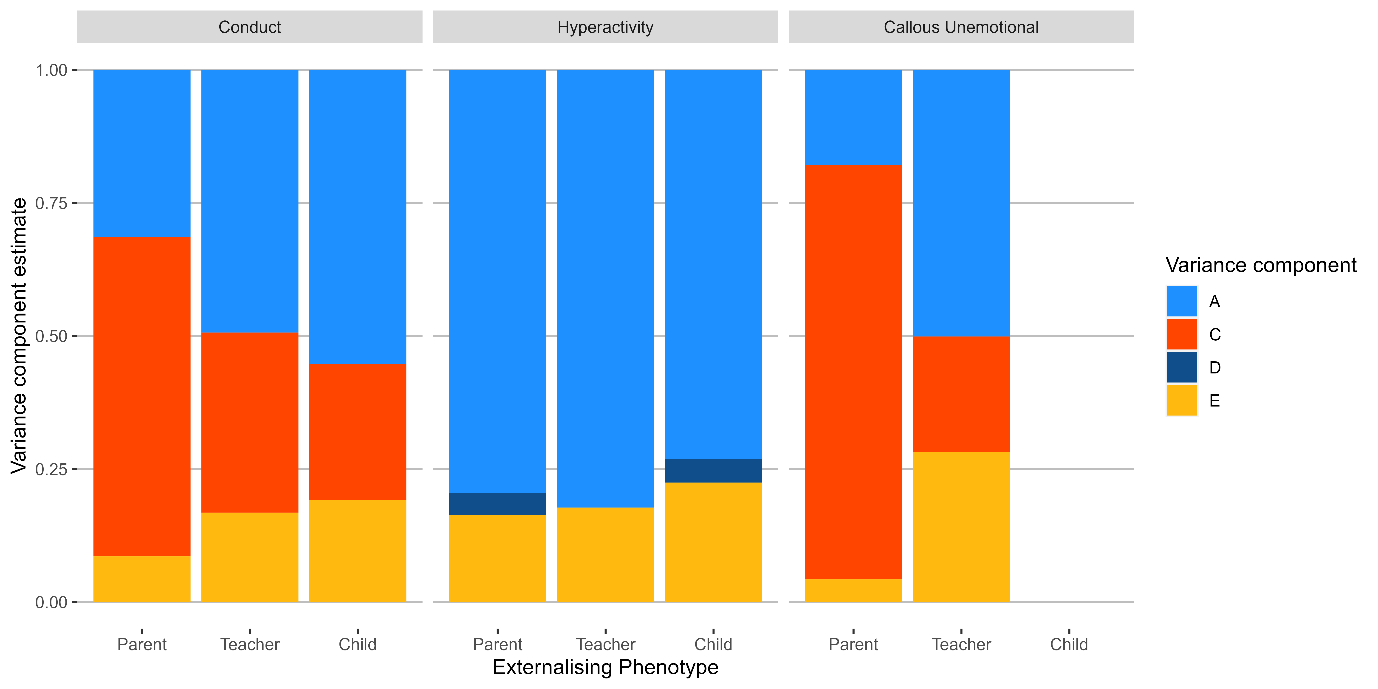
Supplementary Figure 4: Estimating a) population-level, direct and indirect genetic effects of externalising PRS and b) univariate ACE twin models on common factor scores for each phenotype***, ***split by reporter***

Factor scores were created using common factor analysis in lavaan, extracting stability for each phenotype from measures across all timepoints, per reporter to assess whether any of the PRS prediction was driven by a specific reporter. Beta coefficient estimates of population-level prediction of externalising PRS alongside estimates of direct and indirect genetic effects, for common factor scores created for conduct problems, ADHD symptoms and callous-unemotional traits, Estimates of the contribution of additive genetic effects (A), common environmental influences (C) and unique environmental influences (E) in the variance of each factor, per reporter to assess whether estimates of influences on the variance per common factor differed across reporters.

a)

b)

***
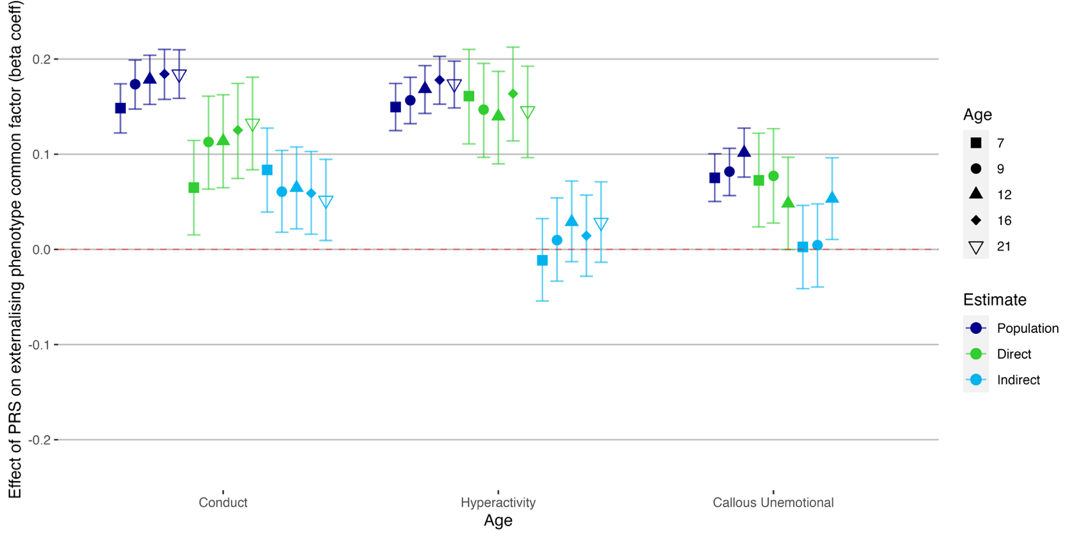
Supplementary Figure 5: Estimating a) population-level, direct and indirect genetic effects of externalising PRS and b) univariate ACE twin models of common factor scores for each externalising phenotype***, ***split by age***

a)

b)


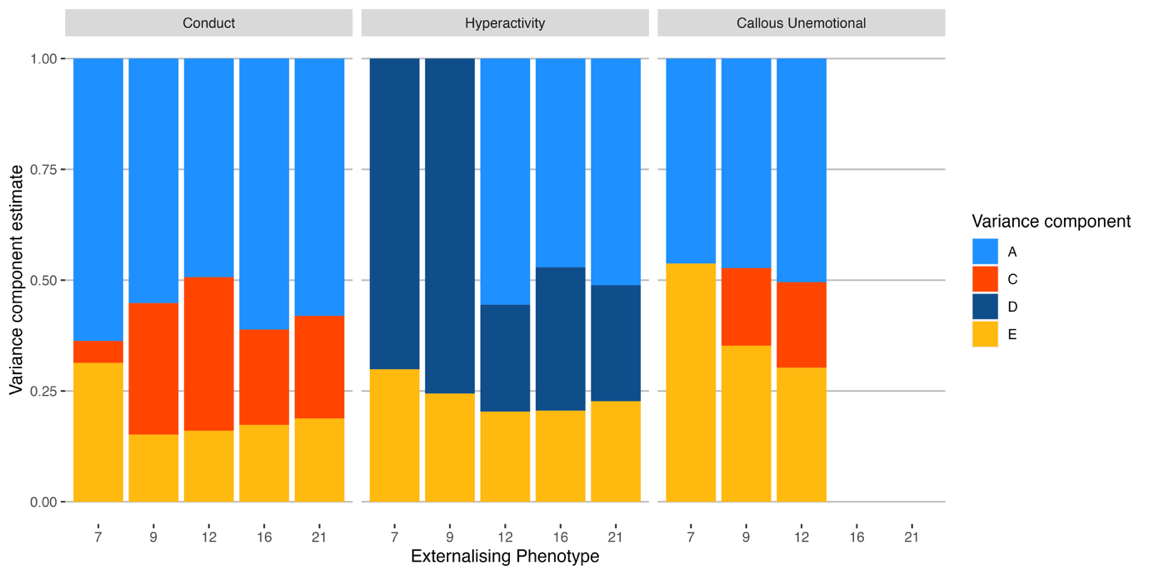

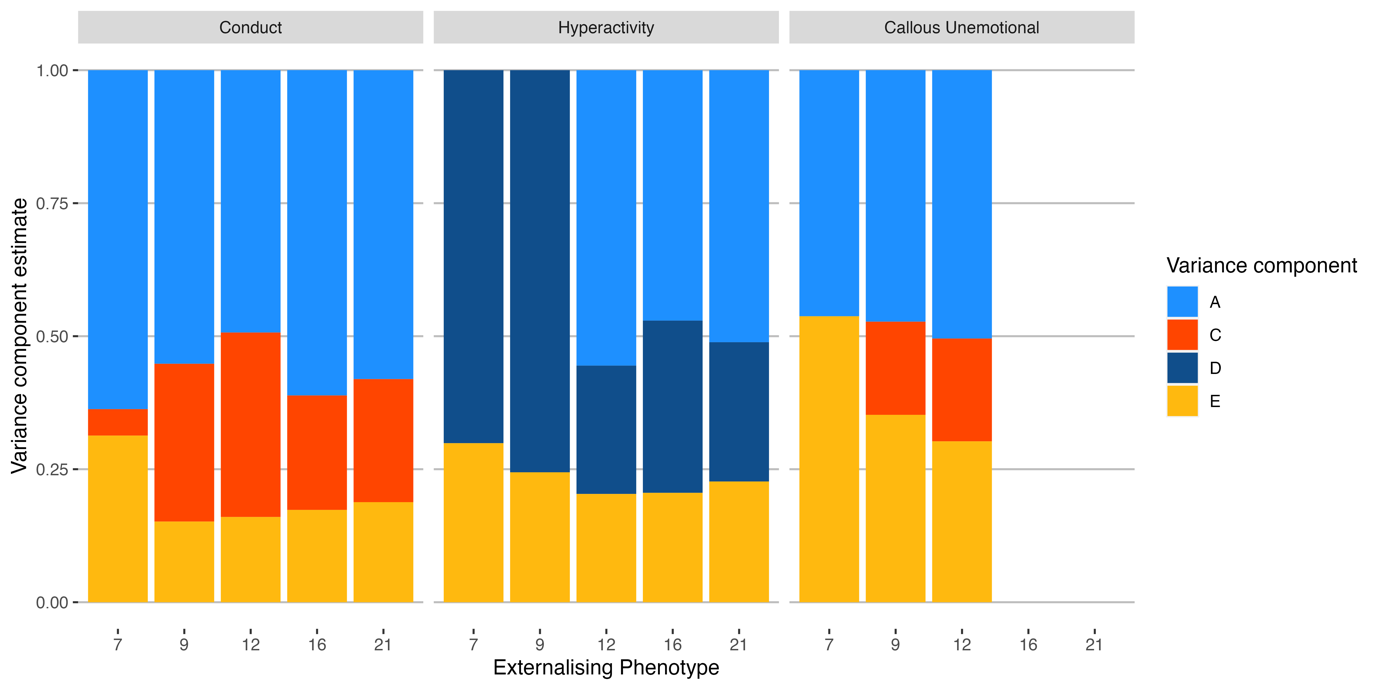


Factor scores were created using common factor analysis in lavaan, extracting stability for each phenotype from measures across all reporters, to assess whether any of the PRS prediction was driven by externalising symptoms at a specific age during development. Beta coefficient estimates of population-level prediction of externalising PRS alongside estimates of direct and indirect genetic effects, for common factor scores created for each externalising phenotype. Estimates of the contribution of additive genetic effects (A), common environmental influences (C), dominant genetic effects (D) and unique environmental influences (E) in the variance of each factor.

***Supplementary Figure 6: Investigating impact of socioeconomic status indices or parenting variables on direct and indirect genetic effects of externalising PRS on a) within-reporter common factor and b) within-timepoint common factor for conduct problems.***

Socioeconomic status (SES) was measured at first contact and comprises measures of parent employment, education, and age of mother on first birth. The Indices of Multiple Deprivation (IMD) decile score uses census data matched with participants post codes, giving a broader measure of wider environmental factors such as local levels of employment and education, crime rates, barriers to housing and living environment quality. The parenting analyses used a latent factor created from ‘Parental Feelings’ and ‘Parental Discipline’ rated by parent at ages 4, 7, 9 and 12.

a)

***
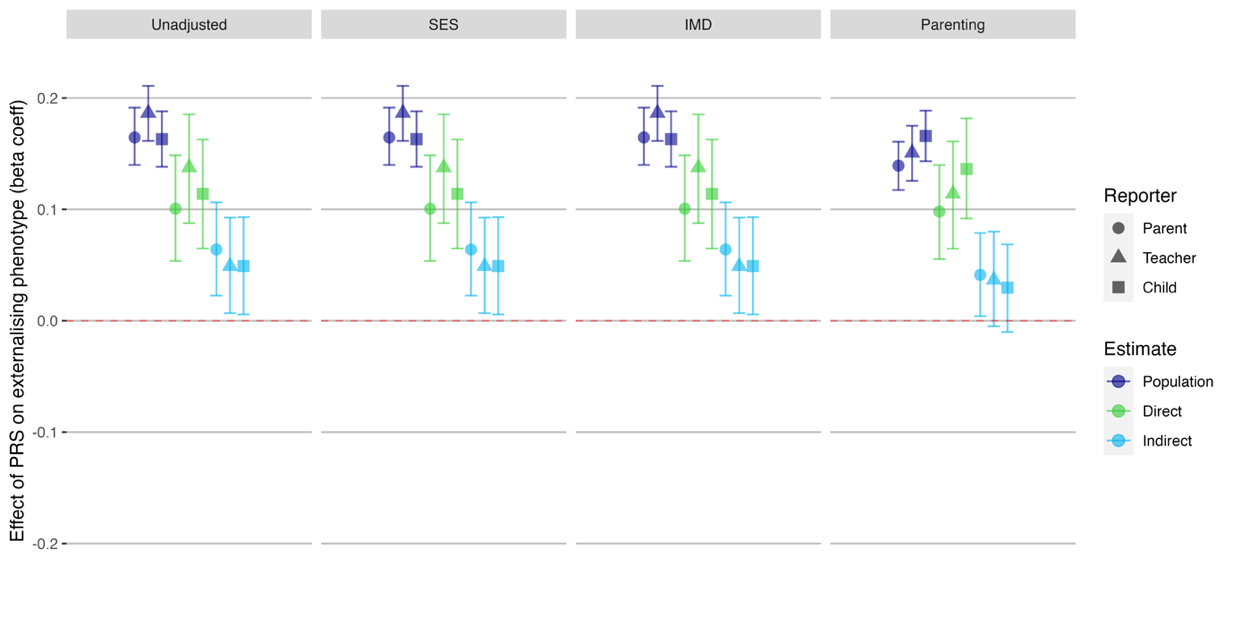

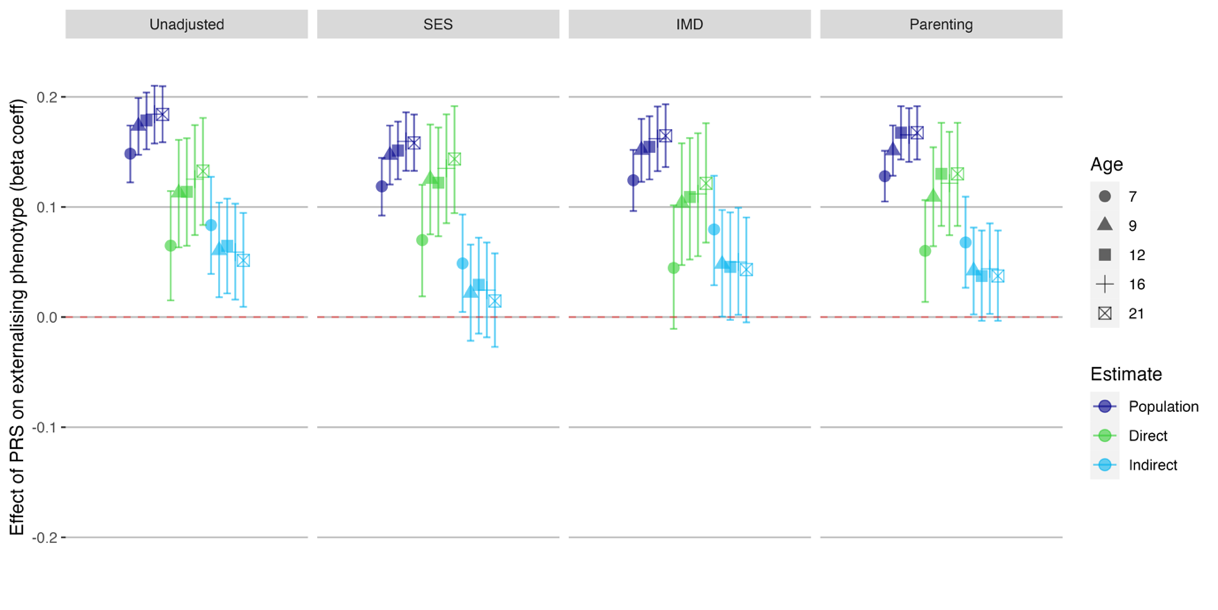
***

b)

***Supp Figure 7: Sensitivity analysis excluding age-9 items from factor score for conduct problems to assess impact of reduced sample size.***

The TEDS sample at age 9 was half the size of other timepoints due to funding and operational constraints. We ran a sensitivity analysis with the full factor score for conduct problems excluding the age-9 items, to investigate the impact on the finding of significant indirect genetic effects.

**
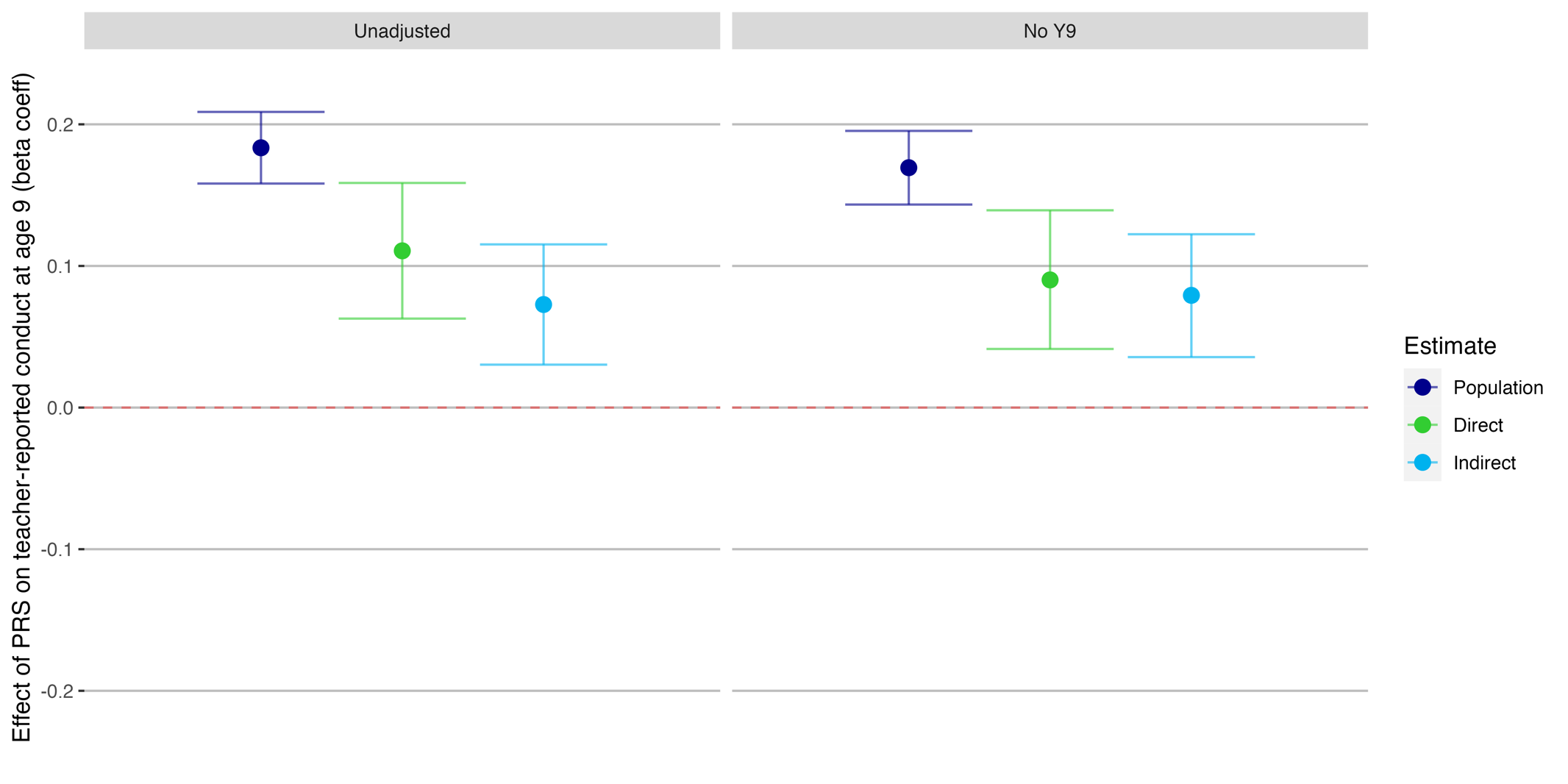
**

**Supplementary References**

1. Takahashi Y, Pease CR, Pingault JB, Viding E. Genetic and environmental influences on the developmental trajectory of callous-unemotional traits from childhood to adolescence. J Child Psychol Psychiatry. 2021 Apr;62(4):414–23.

2. Frick PJ, Hare RD. Antisocial process screening device. Eur J Psychol Assess. 2001;

3. Frick PJ. Inventory of callous–unemotional traits. PLoS One. 2004;

4. Colman AM, Norris CE, Preston CC. Comparing rating scales of different lengths: Equivalence of scores from 5-point and 7-point scales. Psychol Rep. 1997;80(2):355–62.

5. Privé F, Arbel J, Vilhjálmsson BJ. LDpred2: better, faster, stronger. Bioinforma Oxf Engl. 2021 Apr 1;36(22–23):5424–31.
